# Genome-wide association study for crown rust (*Puccinia coronata* f. sp. *avenae*) and powdery mildew (*Blumeria graminis* f. sp. *avenae*) resistance in an oat (*Avena sativa*) collection of commercial varieties and landraces

**DOI:** 10.3389/fpls.2015.00103

**Published:** 2015-03-05

**Authors:** Gracia Montilla-Bascón, Nicolas Rispail, Javier Sánchez-Martín, Diego Rubiales, Luis A. J. Mur, Tim Langdon, Catherine J. Howarth, Elena Prats

**Affiliations:** ^1^Institute for Sustainable Agriculture – Consejo Superior de Investigaciones CientíficasCórdoba, Spain; ^2^Institute of Biological, Environmental and Rural Sciences, University of AberystwythAberystwyth, UK

**Keywords:** association analysis, drought, oat, powdery mildew, resistance, crown rust

## Abstract

Diseases caused by crown rust (*Puccinia coronata* f. sp. *avenae*) and powdery mildew (*Blumeria graminis* f. sp. *avenae*) are among the most important constraints for the oat crop. Breeding for resistance is one of the most effective, economical, and environmentally friendly means to control these diseases. The purpose of this work was to identify elite alleles for rust and powdery mildew resistance in oat by association mapping to aid selection of resistant plants. To this aim, 177 oat accessions including white and red oat cultivars and landraces were evaluated for disease resistance and further genotyped with 31 simple sequence repeat and 15,000 Diversity Arrays Technology (DArT) markers to reveal association with disease resistance traits. After data curation, 1712 polymorphic markers were considered for association analysis. Principal component analysis and a Bayesian clustering approach were applied to infer population structure. Five different general and mixed linear models accounting for population structure and/or kinship corrections and two different statistical tests were carried out to reduce false positive. Five markers, two of them highly significant in all models tested were associated with rust resistance. No strong association between any marker and powdery mildew resistance at the seedling stage was identified. However, one DArT sequence, oPt-5014, was strongly associated with powdery mildew resistance in adult plants. Overall, the markers showing the strongest association in this study provide ideal candidates for further studies and future inclusion in strategies of marker-assisted selection.

## INTRODUCTION

Oat is a grain crop of Mediterranean origin used for both human food and animal feed as well as a green or conserved fodder and, more recently, as a winter cover crop in no-till rotations (Stevens et al., 2004). *Avena sativa* L. including both white and red oat (formerly *A. byzantina* K. Koch) is the main cultivated oat. Several pathogenic fungi can infect oats and drastically reduce its yield including biotrophic pathogens such as powdery mildew (*Blumeria graminis* f. sp. *avenae* Em. Marchal) and crown rust (*Puccinia coronata* f. sp. *avenae* Eriks). These fungi have very efficient spreading mechanisms, hampering their control by crop management measures such as rotation and the use of resistant varieties is one of the best control alternatives (Stevens et al., 2004).

Genetic markers have proved useful for the identification of quantitative trait loci (QTL) associated with important agronomic traits using a number of experimental bi-parental oat populations. Examples include vernalization response, flowering, and heading date (Maloney et al., 2011), quality traits, including seed tocopherol (Jackson et al., 2008), groat protein and oil content (Zhu et al., 2004; Hizbai et al., 2012) and resistance to stresses including winter field survival (Maloney et al., 2011), *Fusarium* resistance (He et al., 2013), powdery mildew resistance (Yu and Herrmann, 2006), and crown rust resistance (Wight et al., 2004; Portyanko et al., 2005; Jackson et al., 2010). However, there are often limitations in the use of such QTL in marker-assisted selection (MAS) as the parental genotypes used in these studies are often not representative of the germplasm pool that is actively used in breeding programs and markers linked to QTL are not always transferable to other genetic backgrounds (Snowdon and Friedt, 2004).

Association analysis is an alternative approach that overcomes many of the limitations of conventional QTL mapping and has received increasing attention from plant geneticists during the last few years (Kraakman et al., 2004; Gupta et al., 2005; Breseghello and Sorrells, 2006; Stracke et al., 2009) following its success in dissecting human diseases (Klein et al., 2005; Cordell et al., 2013; Lee et al., 2013). Association analysis relies on unrelated individuals to create population-wide marker-phenotype associations (Jannink et al., 2001) and is based on linkage disequilibrium, defined as the non-random association of alleles at two loci (Falconer and MacKay, 1996). Linkage disequilibrium among loci is a complex phenomenon, since it is affected by mutation history, population structure, admixture among populations, natural and artificial selection (including breeding), genetic drift, and the organism’s own reproductive biology (Flint-Garcia et al., 2003; Newell et al., 2011). Association analysis utilizes historic patterns of recombination that have occurred within a sample of individuals to detect correlations between genotypes and phenotypes within these individuals (Zondervan and Cardon, 2004).

In recent years, genome wide association studies (GWAS) have identified marker-trait associations for a range of agronomic traits in many crops including maize, rice, sorghum, and foxtail millet (Huang et al., 2010; Jia et al., 2013; Li et al., 2013; Morris et al., 2013). However, there are fewer reports of the use of GWAS with stress resistance traits although marker associations with disease resistance have been identified in maize, rice, and wheat (Kump et al., 2011; Gurung et al., 2014; Wang et al., 2014). In oats, only a few association analysis studies have been reported (Achleitner et al., 2008) and they have primarily focussed on grain quality traits such as beta-glucan concentration (Newell et al., 2012; Asoro et al., 2013) and none have attempted to identify marker-trait associations with some of the most important biotic constraints of this crop, namely powdery mildew and rust pathogenic fungi. In this work, we performed an association analysis in an oat collection of commercial cultivars and landraces based on simple sequence repeat (SSR) and Diversity Arrays Technology (DArT) genotyping following a detailed study of population structure and linkage disequilibrium and identified several markers associated with rust and powdery mildew resistance.

## MATERIALS AND METHODS

### PLANT MATERIAL

For this study, a germplasm collection of landraces consisting of 141 *A. sativa* accessions (110 white and 31 red oats) kindly provided by the “Centro de Recursos Fitogenéticos,” INIA, Madrid, Spain, and 36 commercial varieties supplied by the Andalusian Network of Agriculture Experimentation (RAEA) was used. Oat cultivars studied were: Ac1, Acebeda, Adamo, Aintree, Alcudia, Anchuela, Araceli, Brawi, Caleche, Cannele, Chambord, Chappline, Charming, Cobeña, Condor, Cory, Edelprinz, Flega, Fringante, Fuwi, Hammel, Kankan, Kantora, Karmela, Kassandra, Kazmina, Mirabel, Mojacar, Norly, Orblanche, Pallini, Patones, Prevision, Primula, Rappidena, and Saia. Details of the origin of all accessions and of their genetic relationship have been previously reported in Montilla-Bascón et al. (2013).

Seedlings were grown in 0.5 L pots filled with peat:sand (3:1) in a growth chamber with 20°C, 65% relative humidity (RH) and under 12 h dark/12 h light with 250 μmol m^-2^ s^-1^ photon flux density supplied by high-output white fluorescent tubes.

### GENOTYPING AND DATA CURATION

First leaves from 40 12-days-old seedlings were harvested, pooled together, and DNA extracted according to the method stipulated by Diversity Arrays P/L, Canberra, ACT, Australia and described by Tinker et al. (2009). SSR analysis was as previously described Montilla-Bascón et al. (2013). SSRs used were chosen for their amplification consistency and polymorphism in our oat genotypes and/or because they displayed reasonable genome coverage in a mapping population developed from the winter oat cultivars Buffalo and Tardis (C. J. Howarth personal communication). DArT marker analysis using the high density oat array (15,000 markers) was performed by Diversity Arrays P/L, as described in Tinker et al. (2009).

To remove possible errors and redundancies in markers that may cause false associations in GWAS, data cleaning was performed according to Miyagawa et al. (2008). Markers with >20% missing data were removed as were those with a minor allele frequency (MAF) of less than 1%. Markers that diverged less than 1% across the genotypes lines were merged, thus combining markers that were in near perfect LD. Finally, inspections were performed to determine accessions that differed by less than 1% to remove any redundant accessions, however, in our study no accessions fell into this category.

### GENETIC DISTANCE, POPULATION STRUCTURE, AND KINSHIP

Estimates of genetic distance were calculated according to Nei and Li (1979) parameter with Arlequin software. Population structure was inferred by the software STRUCTURE 2.3.4 (Pritchard et al., 2000) using the admixture model and the option of correlated allele frequencies between populations. Similarly, the degree of admixture alpha was inferred from the data. Each simulation included 20,000 burn-in and 100,000 iterations. Longer burn-in or MCMC did not change significantly the results. Ten independent simulations per *k*-value were run. Then, the mean estimate across runs of the log posterior probability of the data for a given *k*, were plotted to enable the determination of the *k*-value of the population. As this point is known to be difficult to determine, the Δk, related to the second order rates of change of the likelihood function with respect to *k*, was also used (Evanno et al., 2005). The percentages of admixture of each accession (Q matrix) given by the software were used as cofactors in the association analyses. For trait analyses per subpopulation, an accession was assigned to a subpopulation when it showed more than 80% membership in this subpopulation (de Alencar Figueiredo et al., 2010). Principal component analysis (PCA) was also performed as an alternative method to infer the structure of the collection with the software package PAST (Hammer et al., 2001).

The kinship coefficient approach proposed by Yu et al. (2006) allows taking possible family relatedness into account and can help removing additional false positives. These coefficients (K matrix) were computed with the software TASSEL 4.1.27 (Bradbury et al., 2007).

### LINKAGE DISEQUILIBRIUM

Linkage disequilibrium measured as *r^2^* was calculated by software TASSEL 4.1.27 for each marker pair together with the significance of the parameter. *r^2^* was used, since it is only moderately influenced by small sample sizes and low allele frequencies (Flint-Garcia et al., 2003) and it is relevant for QTL mapping since it relates the amount of variance explained by the marker to the amount of variance generated by the associated QTL (Zhu et al., 2008). The disequilibrium matrix summarizing pair-wise measures of LD was also performed with the software TASSEL.

### PHENOTYPING

#### Crown rust resistance assessment

The *P. coronata* f. sp. *avenae* (*Pca*) isolate Co-04, previously multiplied on the susceptible cultivar Araceli, was used. The virulence of this isolate on an oat differential set collection has been described in Sánchez-Martín et al. (2012). Four independent plants per accession were grown in a growth chamber as described above and when the first leaf had fully expanded they were inoculated with urediospores mixed with pure talcum powder (1:1, w/w) by dusting them over the plants to give approximately 30 spores mm^-2^ (checked by counts made from glass slides laid adjacent to leaves). After inoculation, plants were incubated for 9.5 h in darkness at 100% RH and 18°C, and thereafter at 20°C under a 12 h photoperiod with 250 μmol m^-2^ s^-1^ photon flux density. Infection frequency (IF) was determined as previously described in Prats et al. (2002). IF scores were converted into relative infection frequency (RIF) values expressed as the percentage of the susceptible reference cultivar Araceli.

#### Powdery mildew resistance assessment

Four independent plants per accession were grown as described above and when the first leaf was fully expanded it was inoculated using a settling tower (Lyngkjær et al., 1997) to give about 30 conidia mm^-2^ with one isolate of *B. graminis* f. sp. *avenae* race five maintained on seedlings of oat cv. Selma, in a spore proof glasshouse. After inoculation, plants were maintained in the growth chamber for 8 days before assessment of the percentage area covered by powdery mildew on the inoculated leaf. Disease scores were converted into relative values, expressed as the percentage of the susceptible reference cultivar Selma and referred to as the relative disease severity (RDS; Rubiales et al., 1993; Martínez et al., 2007). For assessment of adult plant resistance the fifth leaves were inoculated and macroscopically assessed as above without excising the leaves from the plant.

### STATISTICAL ANALYSES

For phenotype assessments the experimental design was arranged according to randomized complete block design with four independent blocks each containing the whole set of accessions randomly ordered. For ease of understanding, means of raw percentage data are presented in tables and figures. However, for statistical analysis, data recorded as percentages were transformed to arcsine square roots (transformed value = 180/Π × arcsine [√(%/100)]) to normalize data and stabilize variances throughout the data range, and subjected to analysis of variance using SPSS software, after which residual plots were inspected to confirm that data conformed to normality. Significance of differences between means was determined by contrast analysis (Scheffe’s). The percentage of variation of each trait explained by the structure was computed through multiple linear regression of the phenotypes on the percentages of admixture using R (Ihaka and Gentleman, 1996).

### ASSOCIATION ANALYSIS

Associations between molecular markers and phenotypes were computed using the software package TASSEL 4.1.27 (Bradbury et al., 2007). Five models were used: a simple general linear model (GLM), a GLM model using the percentages of admixture of each accession (Q matrix) as cofactors to take population structure into account (GLM-Q), a GLM model using the PCAs covariates as cofactors (GLM-PCA), a GLM model using both Q matrix and PCAs covariates (GLM-Q-PCA) and a mixed linear model (MLM) using both the percentages of admixture and the kinship coefficients as cofactors (Q and K matrices). All GLM procedures tested fixed-effect models in which mean phenotypes of a given trait were predicted by the independent variables. Tests were run with 1,000 permutations allowing determination of the site-wise *p*-value for each marker, which is the probability of a greater *F*-value under the null hypothesis that the polymorphic site is independent of phenotype. All models were assessed for their ability to control for type I error by plotting the distribution of the *p*-values for the markers, where uniformly distributed *p-*values indicate proper control for type I errors (Newell et al., 2012). The Benjamini and Hochberg (1995) false discovery rate (FDR) criteria at *q =* 0.25 was used to control for multiple testing (Newell et al., 2012) after estimation of the *q-*values of each *p-*values with the module QVALUE (Storey, 2002) in the R v2.15.2 package.

### SEQUENCE HOMOLOGY

As many of the DArT markers used here have been previously sequenced (Tinker et al., 2009), the NCBI non-redundant protein database (database released on 11 January 2015) was searched using the function BlastX of the BLAST algorithm (Altschul et al., 1990) implemented in the NCBI webserver () to further characterize the most significant markers identified.

## RESULTS

### DATA CURATION

Of the initial 15,000 DArT markers assessed, 1,587 showed polymorphism in the oat collection. In addition 499 SSR alleles were also polymorphic. From the total 2086 polymorphic markers, 11 markers that showed a call rate lower than 80% and 56 markers that showed a MAF < 0.01 were removed. A total of 476 redundant markers were also merged in 169 groups representing these markers. Following data curation a total of 1,712 markers were used for association purposes in the oat collection of 177 white and red commercial varieties and landraces.

### STRUCTURE OF THE POPULATION

**FIGURE 1:**
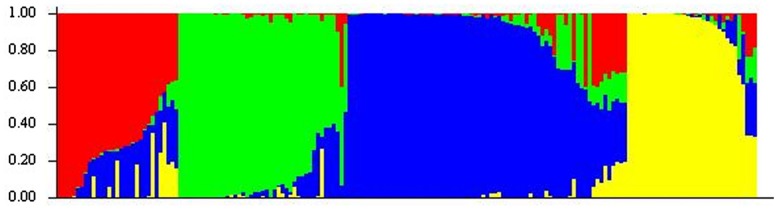
**Estimated population structure of oat genotypes according to STRUCTURE software.** Each individual is represented by a thin vertical segment, which can be partitioned into four colored segments that represent the individual estimated membership to the four clusters.

A previous genetic diversity study of the oat collection with only SSR markers revealed a structure of four subpopulations (Montilla-Bascón et al., 2013). In the present study the number of markers was increased to more than 1,500 and STRUCTURE software indicated the same number of subpopulations (**Figure 1**). Indeed, the correlation between SSR and DArT+SSR results was high with a correlation coefficient of 0.84 (*p* < 0.001). However, slight modifications of the genotype-cluster assignation and the corresponding percentage of admixture were observed. According to both analyses ∼30% of the accessions showed less than 80% of membership for a particular cluster. The differences of genotype-cluster assignation were always related to these accessions and when they were discarded from the analysis the correlation coefficient increased up to 1. According to STRUCTURE, subpopulation 1 showed the highest degree of admixture with 75.6% of the genotypes with less than 80% of membership to this subpopulation followed by subpopulations 3 and 4 with 18% of genotypes with less than 80% of membership in these groups. Subpopulation 2 in which only 15% of genotypes showed less than 80% membership to the corresponding subpopulation was the subpopulation with lowest admixture.

**FIGURE 2:**
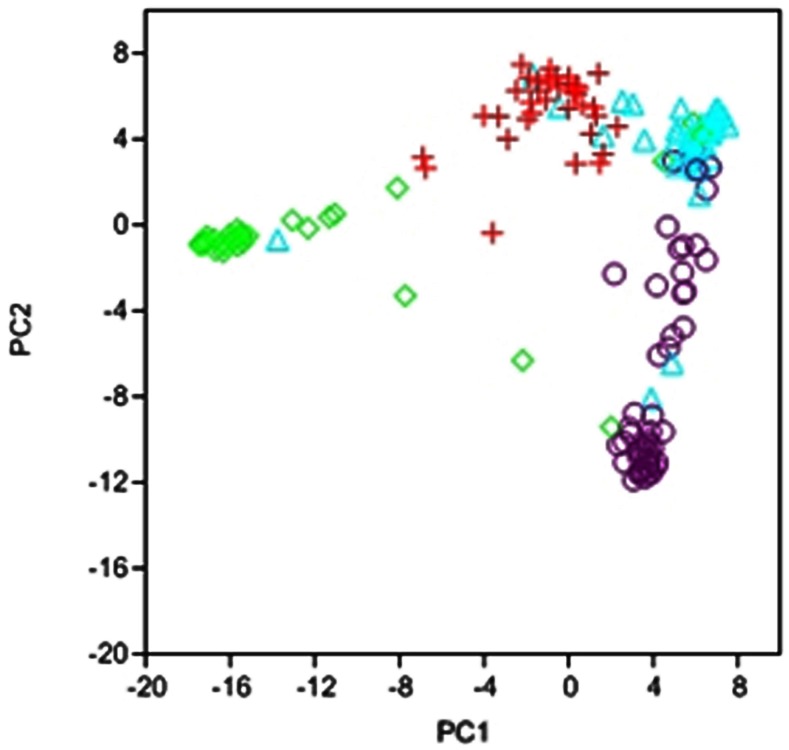
**Scatterplot of Principal Component Analysis scores of components 1 and 2 based on 1712 DArT and SSR markers used in this study.** Represented are the genotypes belonging to cluster 1 (red), cluster 2 (green), cluster 3 (violet), and cluster 4 (blue).

Multivariate analysis based on PCA also revealed a separation of four subpopulations which indicates a high consistency of the data (**Figure 2**). Cluster analysis was implemented on the first four principal components cumulatively explaining ∼50% of the variation with 23.3, 13.8, 8.11, and 4.5% for each of the components, respectively. Although separation between clusters was clear, some accessions were not part of the clusters but formed links between them (**Figure 2**). The number of lines in each cluster ranged from 33 to 64. The first cluster included mainly the white commercial varieties, cluster 2 the red oats, cluster 3 the white oat landraces characteristic to high altitude locations, and cluster 4 white oat landraces more adapted to low altitude locations.

### CLUSTER RELATIONSHIPS

As previously stated, PCA showed a separation between clusters but also a clear pair-wise relationship between clusters. Quantitative results for genetic distance (according to Nei’s parameter) between clusters are shown in **Table 1**. Cluster 2 comprising the red oats was by far the most distant from all other clusters, with an average distance of 180 whereas the two white oat landrace clusters were the most closely related groups with an average distance of 66. Clusters 1 and 4 corresponding to the white oat landraces adapted to low altitude and the commercial varieties, respectively were also closely related with a distance of 75 (**Table 1**). These relationships between clusters were in agreement with those depicted by the PCA scatter plot (**Figure 2**). These results suggest that clustering was also efficient in separating the oat types for the germplasm used in this study.

**Table 1:** Population average pair-wise genetic distance according to Nei’s parameter of pair-wise difference.

	C1	C2	C3
C2	150		
C3	125	205	
C4	75	185	66

### LINKAGE DISEQUILIBRIUM

**FIGURE 3:**
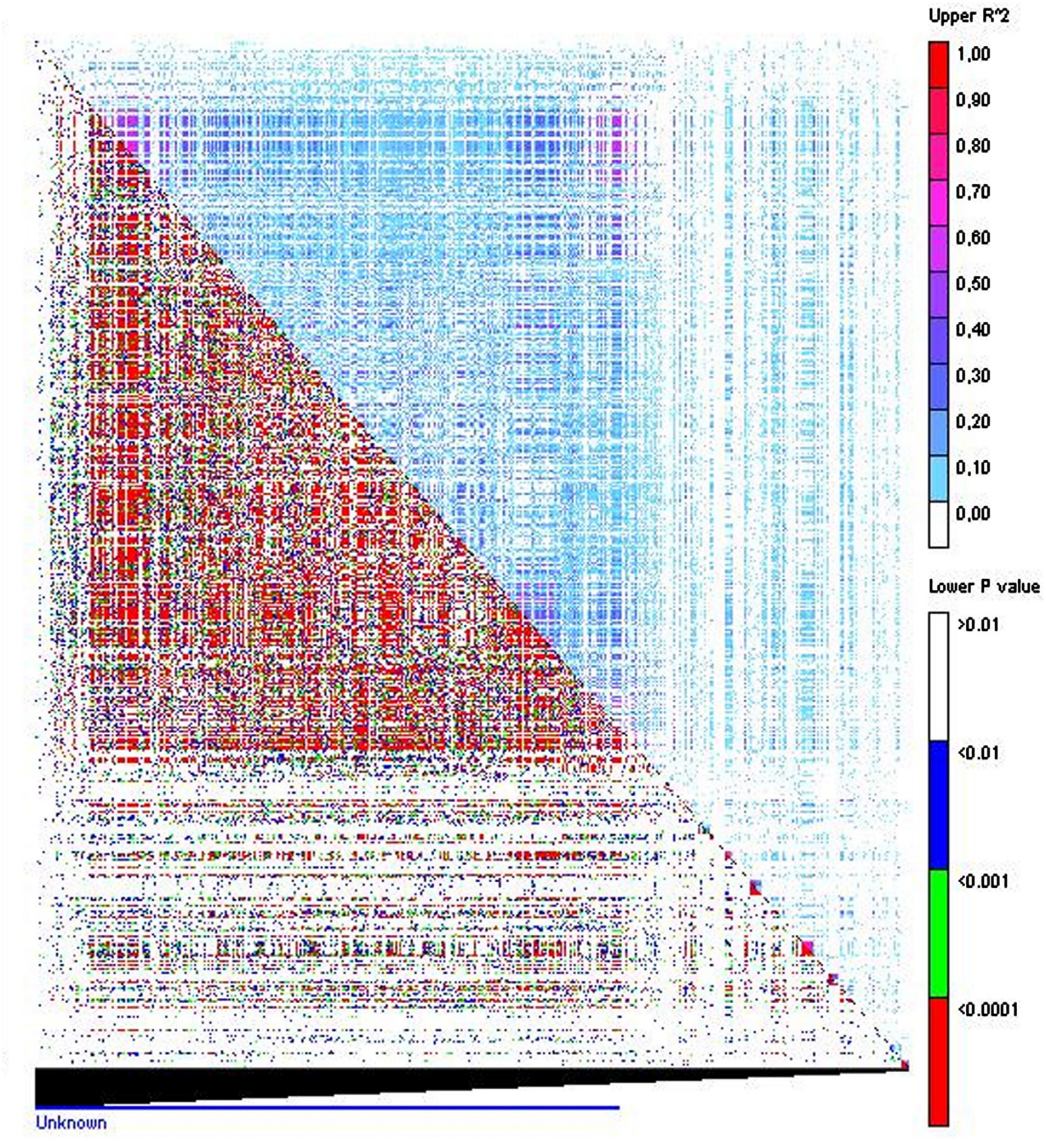
**Linkage disequilibrium matrix.** Pair-wise LD values of polymorphic sites displaying *r^2^* above the diagonal and the corresponding *p-*values from rapid 1000 shuﬄe permutation test below the diagonal. Each cell represent the comparison of two pairs of marker sites with the color codes for the presence of significant LD. Colored bar code for the significance threshold levels in both diagonals is shown.

Identification of disequilibrium between markers is highly useful since it may condition the strength of the association study. Since physical map distances between markers were not available, LD was represented by the disequilibrium matrix visualizing the linear arrangement of LD between polymorphic sites, represented by *r*^2^, and the probability (Flint-Garcia et al., 2003; Gaut and Long, 2003; **Figure 3**). A total of 507,042 pairs of markers showed a significant LD value with an average *p* = 0.004. From these, 277,920 pairs of markers showed an *r*^2^ < 0.1 chosen here as nominal level, according to the studies performed by Newell et al. (2011) in oat. LD of each cluster showed similar values with the exception of cluster 2 that showed a slightly higher LD, probably reflecting the low number of individuals of this cluster of red oats.

### PHENOTYPIC DATA

**FIGURE 4:**
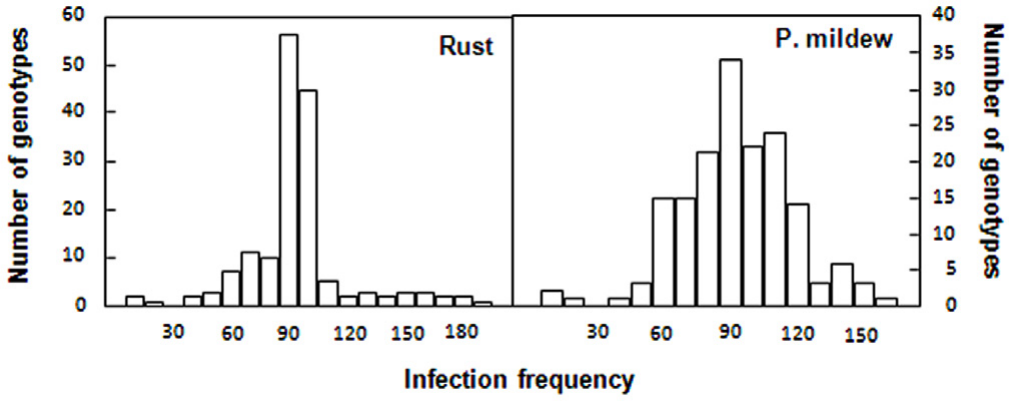
**Distribution of the infection frequency of the rust and powdery mildew infection in the oat collection.** Infection frequency recorded as number of pustules per unit area for rust and percentage of leaf covered by mycelium for powdery mildew were converted into relative values, expressed as percentage of the reading of the susceptible controls, respectively.

Both traits followed a normal distribution with accessions ranging from highly resistant to highly susceptible (**Figure 4**). Means of the 177 accessions assigned to the four subpopulations for the different traits, excluding the admixed accessions were compared (**Table 2**). Significant differences between subpopulations were observed for all traits. Thus, subpopulation 4 had a significantly lower RIF after rust inoculation than the others (*p* < 0.005) and showed a high resistant response. Subpopulation 2 had lower RDS to powdery mildew than the others (*p* < 0.001; **Table 2**). Powdery mildew was the trait most affected by population structure although the proportion of variance explained by population structure remained under 4% (**Table 3**).

**Table 2:** Mean comparison between subpopulations for the accessions assigned to a subpopulation (>80% membership in the subpopulation).

Subpopulation	Rust^a^	PM^b^
1	90.1^ab^	88.6^a^
2	108.8^a^	72.9^b^
3	95.5^ab^	90.8^a^
4	86.0^b^	86.1^a^

*^a^Relative infection frequency (number of pustules cm^-2^) 8 days after rust inoculation; ^b^PM (powdery mildew) resistance expressed as relative disease severity (percentage of the leaf covered by mycelium) 8 days after powdery mildew inoculation*.

**Table 3:** Statistic for stress resistance and percentage of variation of these traits explained by population structure (*K* = 4) through multiple linear regression.

Trait	Mean	Minimum	Maximum	SD	CV (%)	Variance (%)
Rust^a^	89.57	2.05	196.40	28.51	31.83	1.5^ns^
PM^b^	85.51	0.00	150	27.44	32.09	3.7*

*^a^Relative infection frequency (number of pustules cm^-2^) 8 days after rust inoculation; ^b^PM (powdery mildew) resistance expressed as relative disease severity (percentage of the leaf covered by mycelium) 8 days after powdery mildew inoculation*.

### ASSOCIATION ANALYSIS

**Tables 4** and **5**shows the markers considered to be significantly associated with rust or powdery mildew resistance traits according to the threshold of 0.25 for *Q*-value in the FDR test (Newell et al., 2012) in any of the models corrected for population structure.

**Table 4:** Markers associated with rust resistance according to different models: general lineal model (GLM) corrected for population structure according to percentage of admixture coefficients (Q), principal component covariates (PCA), and mixed lineal model (MLM), corrected with kinship and structure matrices.

Marker	GLM+Q			GLM+PCA			MLM		
	p	FDR	r^2a^	p	FDR	r^2^	p	FDR	r^2^
oPt-11795	1.6⋅10^-7^	**2.6⋅10^-**4**^**	0.18	3.7⋅10^-7^	**6.0⋅10^-**4**^**	0.16	1.6⋅10^-7^	**2.7⋅10^-**4**^**	0.22
MAMA5-163	3.2⋅10^-4^	**0.11**	0.08	1.9⋅10^-4^	**0.10**	0.08	1.3⋅10^-4^	**0.11**	0.10
AM30-178	3.5⋅10^-5^	**0.02**	0.10	9.4⋅10^-5^	**0.07**	0.09	1.0⋅10^-3^	0.49	0.07
AME176-3	4.8⋅10^-4^	**0.11**	0.09	8.0⋅10^-4^	**0.25**	0.08	1.2⋅10^-3^	0.49	0.09
oPt-15665	7.0⋅10^-3^	0.42	0.06	9.8⋅10^-4^	**0.20**	0.08	2.9⋅10^-3^	0.72	0.08

*Markers were considered to be associated with the traits if the markers were significant (FDR < 0.25) in GLMs corrected for population structure or MLM. Data in bold indicates values statistically significant according to the false discovery rate (FDR) test*.

*^a^Percentage of phenotypic variance (partial *r^2^* × 100%) of the total variation explained by the marker after fitting the other model effects*.

**Table 5:** Markers associated with powdery mildew resistance according to different models: general lineal model (GLM) corrected for population structure according to percentage of admixture coefficients (Q), principal component covariates (PCA), and mixed lineal model (MLM), corrected with kinship and structure matrices.

Marker	GLM+Q			GLM+PCA			MLM		
	p	FDR	r^2a^	p	FDR	r^2^	p	FDR	r^2^
***Seedling Stage***
oPt-14317	1.5⋅10^-4^	**0.25**	0.10	3.8⋅10^-4^	0.45	0.09	8.0⋅10^-4^	0.90	0.09
***Adult Plant Stage***
oPt-5014	7.1⋅10^-6^	**0.01**	0.34	6.7⋅10^-6^	**5.4⋅10^-**3**^**	0.36	3.2⋅10^-4^	**0.19**	0.35
oPt-3306	5.7⋅10^-5^	**0.04**	0.29	7.5⋅10^-5^	**0.04**	0.30	7.7⋅10^-4^	0.62	0.30
oPt-793335	3.5⋅10^-4^	**0.01**	0.24	5.0⋅10^-6^	**5.4⋅10^-**3**^**	0.36	2.1⋅10^-3^	0.99	0.26

*Markers were considered to be associated with the traits if the markers were significant (FDR < 0.25) in GLM corrected for population structure or MLM. Data in bold indicates values statistically significant according to the false discovery rate (FDR) test*.

*^a^Percentage of phenotypic variance (partial *r^2^*x100%) of the total variation explained by the marker after fitting the other model effects*.

As expected, considerably fewer markers showed a significant association with rust resistance when applying a correction accounting for the population structure than when using GLM alone (for simplicity GLM alone and GLM-Q-PCA are not presented in the tables). The low ability of the GLM alone to account for false positives was confirmed when the distribution of observed *p-*values was plotted in the negative log_10_ scale (Figure S2). The distribution of the *p-*values did not fit with the expected values represented by the solid line, indicating an over-abundance of low *p*-values. However, in the models accounting for structure the distribution of *p-*values fitted better with the expectation. Interestingly, population structure correction by PCA was more efficient in removing false positives than by STRUCTURE software for this data set (Figure S2). Five markers significantly associated with rust resistance were found. Markers oPt-11795 and MAMA5-163 were the two most significantly associated showing significant association in all models tested including MLM and explained 20 and 10% respectively of the variation observed for this trait (**Table 4**). This last model showed almost a perfect fit between the observed and expected *p*-values except for few of the values, which is the characteristic of a model that sufficiently accounts for the number of false positives. Three additional markers, AM30-178, AME176-3, and oPt-15665 were significantly associated in all GLM models including those accounting for population structure (GLM-Q, GLM-PCA, and GLM Q+PCA) but not in the MLM models (**Table 4**).

A significant reduction of associated markers with powdery mildew resistance in oat at seedling stage was also observed after correcting for the population structure than when using GLM alone. The low efficiency of the GLM alone in this data set was confirmed by the low fit of the observed and expected distribution of the *p-*values (Figure S3). Since the significance of the oPt-14317 marker association with powdery mildew resistance in seedlings according to GLM-Q model was at the limit of 0.25 (**Table 5**) and since it was not highlighted by the other models it was not considered to be strongly associated. Indeed, distribution of the *p*-values in the GLM model corrected with Q indicated also a relative over-abundance of low *p-*values. Interestingly, the reverse tendency was observed in the MLM with *p*-values moving down the expected values, indicating a scarcity of low *p-*values and indicating that the GLM+PCA or GLM+Q+PCA were the best fitted models. This indicated the importance of testing the models in order to help in the selection of the most robust markers.

In order to find markers associated with powdery mildew resistance we took advantage of a previous detailed evaluation for powdery mildew adult plant resistance performed in a subpopulation of this collection (Sánchez-Martín et al., 2011). In this, following a preliminary field assessment, 54 genotypes representing the different clusters were evaluated under controlled conditions for adult plant resistance. This population covered a continuous range for powdery mildew resistance between 0 and 100%, showed a similar structure to the full oat collection and showed 414,311 significant marker pairs in linkage disequilibrium with 70,657 of them showing a *r^2^* < 0.1 (Figures S1A–C). Association analysis for adult plant resistance yielded a marker, oPt-5014, highly significant in all models tested. This marker explained ∼30% of the observed variation according to *r^2^*. In addition, two other markers, oPt-3306 and oPt-793335, were strongly associated in the GLM performed accounting for population structure, both through Q covariates and PCA. Distribution of the *p*-values in these models and particularly in that taking into account both Q and PCA fitted well with the expectation indicating a good consistency of the markers. Again, with this data set the MLM model seemed to be excessively restrictive in respect to the significance of the markers (Figure S4).

### SEQUENCE HOMOLOGY

Although the markers significantly associated with the observed phenotypes are likely to be non-functional as they have been identified through LD, sequence information is available for many of the DArT markers evaluated (Tinker et al., 2009, Table S1) and analysis with BlastX proved interesting. **Table 6** show the most significant matches found with BLAST searches (BLASTX) against public databases for associated DArT markers. For most DArT markers, no significant match to specific genic sequences was identified with most significant hits corresponding to repetitive sequences and retrotransposons (i.e., oPt-5014). However, moderately significant matches to disease resistance genes, including the wheat rust resistance locus *Lr21*, were seen for a DArT marker flanking MAMA5, oPt-14345 (FI159838, Tinker et al., 2009). In addition, the marker oPt-11795 showed homology with an autophagy-related protein 2 from *Triticum urartu* and marker oPt-15665 with an anthocyanin 5-aromatic acyltranferase of *Aegilops tauschii*.

**Table 6:** Potential homologous sequences of significant markers using the function BlastX of the BLAST algorithm (Altschul et al., 1990).

Marker	Blastx	Species	E-value	Cov(%)	Ident(%)	Accession number
oPt-11795	Autophagy-related protein 2	*Triticum urartu*	1E-12	85	38	EMS54055
	Hypothetical protein	*Oriza sativa*	2E-12	96	38	EEE52488
oPt-15665	Anthocyanin 5-aromatic acyltransferase	*Aegilops tauschii*	1E-23	54	56	EMT29726
	Hypothetical protein	*Sorghum bicolor*	1E-22	65	41	XP_002450696
	Hypothetical protein	*S. bicolor*	3E-17	50	45	XP_002445048
oPt-5014	Hypothetical protein	*S. bicolor*	6E-30	94	61	XP_002465514
	Hypothetical protein	*S. bicolor*	2E-26	90	56	XP_002450843
	Hypothetical protein	*S. bicolor*	5E-26	93	58	XP_002459653
	Uncharacterized protein	*Brachypodium distachyon*	2E-25	93	53	XP_010233076
	Uncharacterized protein	*B. distachyon*	1E-24	98	47	XP_010239298

Blastx 2.2.30. Data of release January 11, 2015 1:49 A.M.

## DISCUSSION

As a first step for the association study, population structure was inferred since it has great implications on the design and analysis of GWAS. The different approaches used here indicated moderate population structure within the germplasm collection evaluated. Thus, four oat groups could be detected albeit they presented a certain degree of admixture according to STRUCTURE software with up to 30% of accessions having less than 80% membership to a determinate group. This was also observed following PCA with several accessions covering “gaps” between clusters. Interestingly the group comprising the commercial varieties showed the highest degree of admixture, most likely due to a sharing of common ancestors in their genealogy as reported by Montilla-Bascón et al. (2013). One particular concern to oats is the existence of potential population structure arising from the different oat types, winter or spring sown, or interbreeding species such as the white and red (formerly *A. byzantina*) oats. Indeed, in a study by Newell et al. (2012) a small cluster of red oat differentiated from the rest of the collection. Taking into account that our collection was consciously formed with diverse oat types to achieve high genetic diversity, its population structure was considered relatively weak compared with that found in other cereals such as barley (Hamblin et al., 2010) or wheat (Stich et al., 2008).

Despite the relative moderate structure of this oat population, it contains a reasonably high genetic diversity, showing ∼10% of polymorphic markers as in the original oat panel (Tinker et al., 2009). This is an important feature in order to find markers significantly associated with a trait (Ingvarsson and Street, 2011). Estimation of genetic distances between accessions and clusters revealed inter- and intra-group genetic diversity which is confirmed by phenotypic evaluation for responses to rust and powdery mildew that showed wide variability in the degree of resistance which extended to complete susceptibility. As expected the less divergent groups were the white oat landraces adapted to high and low altitude followed by commercial cultivars while the most distant group was the red oats.

The extent of LD in a species influences the strength and resolution of GWAS. The study of linkage disequilibrium in the oat collection showed a very high number of marker pairs in significant LD. This indicated high genome coverage with non-associated markers. In oats it has been proposed that a marker every cM (2,000 marker in total) would explain, on average 20% of QTL variance. This is not very different from other self-pollinated cereals such as barley in which LD decay is expected to occur over relatively long map distances compared with allogamous cereals such as maize for which reduction of *r^2^* to 0.15 have been reported to occur within 500 bp (Tenaillon et al., 2001). Our work using 1,712 markers should cover a significant part of the genome although increasing the number and distribution of markers would increase the probability of identifying additional markers in high LD with a QTL.

The molecular marker data set in combination with phenotype evaluation was used to examine linkage-related marker-trait associations. Separating the role of population structure and genetic linkage as causes for marker-trait association remains the greatest challenge in association analysis (Achleitner et al., 2008). The five models used in this study accounted for “Q” (population structure from subpopulations) and/or “K” (genetic similarity in the background from shared kinship) which may be important to identify marker-phenotype associations not related to genetic linkage between markers and QTL. In addition models containing PCA covariates, which may account for some proportion of both “Q” and “K” were also tested. A tentative comparison between the GLM and the MLM models was performed since MLM models that accounts for kinship relationships, such as that described by Yu et al. (2006), might remove more of the structure effect. This point was demonstrated by Brown et al. (2008) in sorghum and Cockram et al. (2008) in barley. In our analysis the MLM was generally excessively restrictive and did not outperform the GLMs with structure covariates. Overall, for our data set the GLM+PCA offered the best control of type I errors. Thus, co-examination of different models and traits can provide an informative summary of the major trends affecting the analysis.

Five markers, two of them found highly significant in all models tested, were associated with rust resistance. No significant similarity was identified by BLASTN or BLASTX against NCBI databases other than with putative repetitive elements or retrotransposons. However, two of these DArT sequences showed similarity to an autophagy-related protein 2 and to an anthocyanin 5-aromatic acyltransferase that have been related to the plant immune defense reaction. Thus, recently an autophagy-related protein 2 *Arabidopsis* mutant, atg2-2, has been reported to have enhanced resistance to powdery mildew (Wang et al., 2011) and expression of an anthocyanin 5-aromatic acyltransferase have been found to be altered in resistant *A. thaliana* ecotypes infected with cucumber mosaic virus (Ishihara et al., 2004). However, further work would be needed to ascertain the relationship between the DArTs markers and these genes.

Despite the wide distribution of powdery mildew resistance in our collection, strong association between any markers and seedling resistance was not detected. It may be possible that the combination of marker density and the phenotypic variation were insufficient. Polymorphisms causing variation for this trait may have been in linkage equilibrium with our markers, and higher marker densities could have uncovered more QTLs. Alternatively, a high number of rare alleles causing variation in seedling powdery mildew resistance in our collection might cause less variation in the data and therefore be undetected. Indeed, rare alleles are a leading hypothesis for the “missing heritability observation” in human association studies (Yang et al., 2010). The low association for this trait could also be due to the development of markers from a genetically narrow set of germplasm in relation to the lines used in this study. However, this is highly unlikely since DArT markers were developed from a panel of 60 accessions of global representation. Interestingly one DArT sequence, oPt-5014, was strongly associated with powdery mildew resistance in adult plants. The strong association observed taking into account the relative low number of accessions evaluated for this trait, suggest that a careful selection of accessions covering a complete range of phenotypic and genotypic variation may be adequate in some cases to find significant associations. Marker oPt-5014 was associated with hypothetical proteins of sorghum, wheat and rice containing a Zinc knuckle domain (pfam14392) which has been detected in several plants transcription factors and might therefore be involved in the regulation of gene expression.

Recent oat maps sharing common markers allow us to locate the DArT markers more specifically within the oat genome (Figure S5). According to Tinker et al. (2009), oPt-11795 marker maps onto KO32 which is equivalent to chromosome 4C in the first physically anchored consensus oat map (Oliver et al., 2011) where there are no previously reported crown rust resistance genes. Recent studies showed synteny between this chromosome and *Brachypodium distachyon* chromosome 4, *Oryza sativa* chromosome 9 and wheat chromosome 5BL where regions controlling disease resistance have been described; QTLs for resistance to the rust fungus *Puccinia brachypodii* have been reported in chromosome 4 of *B. distachyon* (Barbieri et al., 2012), a powdery mildew resistance gene *PmAS846* mapped in wheat chromosome 5BL (Xue et al., 2012) and a locus associated with broad-spectrum resistance to rice blast, *Pi5(t)*, mapped onto rice chromosome 9. MAMA5 is reported by Wight et al. (2003) to map near to the marker cdo53 on KO17 equivalent to chromosome 9D (Oliver et al., 2013). Interestingly, the partial crown rust resistance *Pc38* that cluster with *Pc62* and *Pc63* (Harder et al., 1980) also maps in this position (Wight et al., 2004) together with the major QTL for partial rust resistance, *Prq1b* (Portyanko et al., 2005). AME176 maps onto chromosome 15A (unpublished Buffalo × Tardis results) which shows homology with chromosome 9D where according to Oliver et al. (2013) a number of other resistance genes map. According to Tinker et al. (2009), oPt-14317 maps onto KO22_44_18 within the same framework marker as AM102 now annotated as chromosome 19A. This is a similar position to where the dominant powdery mildew resistance gene *Eg5* has been mapped (Yu and Herrmann, 2006). Finally, oPt-5014 has been mapped in a number of populations (e.g., Hizbai et al., 2012; He et al., 2013) onto chromosome 21D. This chromosome is also known to contain a number of crown rust resistance genes such as *Pc54*, *Pc59,* and *Pc68*. However, lack of common markers makes it difficult to determine how close oPt-5014 is to these genes.

Overall, the markers showing the strongest association in this study provide ideal candidates for further studies and future inclusion in strategies of MAS.

## AUTHOR CONTRIBUTIONS

GM-B, NR, and JS-M carried out most of the experimental work and data analysis. DR contributed to the disease resistance aspects. TL, CH, and LM contributed to the genetic aspects. EP designed experiments, and contributed to the interpretation of results and writing of the manuscript. NR, DR, TL, CH, and LM also contributed to critical reading and writing.

## Conflict of Interest Statement

The authors declare that the research was conducted in the absence of any commercial or financial relationships that could be construed as a potential conflict of interest.
