# Supplementary material for: Genome-wide association study for crown rust (Puccinia coronata f. sp. avenae) and powdery mildew (Blumeria graminis f. sp. avenae) resistance in an oat (Avena sativa) collection of commercial varieties and landraces
Source: Front Plant Sci. 2015 Mar 5;6:103. doi: 10.3389/fpls.2015.00103 (PMC4350391; doi:10.3389/fpls.2015.00103)
Supplement: Supplementary file 1 [file data_sheet_1.pdf]

Supplementary Material

Genome-wide association study for crown rust and powdery mildew resistance in an oat collection of commercial varieties and landraces

Gracia Montilla-Bascón[1#], Nicolas Rispail[1#], Javier Sánchez-Martín[1], Diego Rubiales[1], Luis AJ Mur[2], Tim Langdon[2], Catherine Howarth[2] and Elena Prats[1]*

[1]Institute for Sustainable Agriculture-CSIC, Córdoba, Spain.
[2]Institute of Biological, Environmental and Rural Sciences, University of Aberystwyth, UK

*** Correspondence:** Dr. Elena Prats, Institute for Sustainable Agriculture, CSIC, Apdo. 4084, E-14080 Córdoba, Spain.
elena.prats@ias.csic.es

Supplementary Table 1

Marker	Accession Nº	Sequence
oPt-11795	FI157472	CTGGATTTGTTGTAAACTGATCATCAGTTGGCCGAGTTGA<br>TGTTGGGCAGTTGCTGTCAAAGGAGACAGAAGCTTGAGC<br>ACCTAGTTCCCATCTTCTCGTTGGGATTCTACCGTGCATTG<br>TGGGTCTTCAGCAAAGGAGTATAGTATATCGTAGGCTATG<br>TAAAACCTGCCCCCCTCGGTCCATTTGTCATGCCAGAAAG<br>ATATTTTGGTACCATTTGCCGGGGAGCAGATAGTTGCGCT<br>CTGCACCGTGGGTATCAATGCAGGTAGCGATTTCCAGAAG<br>GGTGTGTCTTGCTGTGATGTTGTGTGTGGTATAGTGCC
oPt-15665	FI158620	CAGGCTAGTAACAAGCAACAAGGGAATATGAAGAAGGGA<br>AAAGTTGTGGTGATTGAAGATCAAAACTCAGGGAGAGAG<br>AATCAGTTAAGGTCAAAAGATAGTTACCTGGATGTGGTCT<br>ACTTTACCTGTGGGGAACCTGGCCATAACAAGTCTCAGTT<br>CCCCTCTGCTCCTTTTTTTTCATATGCAAGATGGTAAACCA<br>CAAGGAAGACAAGTGTCCAGTGAGGAAGCTAGCTTTGCC<br>TGCTGCCAAGCTTTATGGAAAAGCTGCTCAAGGGCTGGAA<br>TTCTTCCATGTGGAGGTTCCTGAAAGCTACAACAATGATA<br>TGGGAGCAAAAAATGTGGGGATTGTGTTCATTGAGGCTG<br>GTGAAATCAACAAAGAGGAGCTGGCTCAGGAGTTTGTAG<br>TTATCTACAAAACTACTTGGCCTTGGCAGATTAGGCAGCT<br>AGATGATTGGTCCTTTCTTGTCAAGTTCCCTCCCCACCTCC<br>CAGTAGAAGATGTAGTTGGTTATCCATGCTTTGGCCTAGT<br>GAAAGATGGGGTTACTGTAAATGTGGAAGTCTAGGATGG<br>GGAA
oPt-14317	FI159214	TGCAGGCAGAGGCAGTGTTTATCCTTGAGGGAGTCCATCC<br>CGAGCAGCGGCATGGCAGCCTGGCCGTTCTGTCCCACTGC<br>CACTGGTATTTCAGCTAGCATCCACAGGCGGCTGGTGTGG<br>GGAGTGCTGGAAATACTAGTCATGTCACCGTCCAGTGTTA<br>GTAGCATTTCTTGAAAGGGAAAAGTCAGCACACACACAC<br>TCTTGGCGCCAGCTCCAGGATCCTCGCTTCTCCACAGGGG<br>GGATGCTGTCGCCAGCTACTAGCTGTAGCCCAGCACTCAC<br>TCACCACTACCCGGGAGTAGGAGTGCGTGATGTTTGCGGC<br>GTGGTGAAAGGTCACATGGAATTTGCCCGATTTGGAGCCT<br>GCATGCCATGCCACGGGGATCATGTGATCTTATTTTTTTGT<br>CTTGCTTTAATTTGAGGAGGAGTTGATGTTACATTACAAT<br>AAGTCAATAATGATAATGATCCGGCCGCGTGGGTATCTTG<br>TGATCCTGGTTGGTGGTTTGTTCCGTTTTGTTCGCATCATC<br>CAAAAACTC

oPt-5014	FI159708	GTCTAGGGGTTAGCGGCTGCAAAGGTACTTACGTCCAAAT<br>CCTTCAGTATGCAGTCGCTAAAGAGCTCAATGCACGCAAC<br>GTGGAATCCAGCAAGGGAAATCTCGTTCACTGCTGCGGA<br>AGAAAATCTGTTCGTACTACAAGCCCAATGTCTAGGGGAC<br>TAGAACTGTATGATGCTGGAGGGCACTTGGCTCTTTAGGG<br>AATGTGCTCTGATGGTTGAACCTTTTGATGGGGCAACAAC<br>GGTGCAGTCATACAGAGAGGTTCCCAAGCATGGGTTCAA<br>ATCCACAAATTACCATCACTATTTTGCAAGAAGCAGGTTC<br>TTGATCAATTGGCAAGCAGAGTGGGAGAACTGATATCTAC<br>TGATTTGACCCCTGTTCAGATGCGTACAGGTGTGTTTCATC<br>GGGTACGCGTGAAGCTTAACTCTGCAAAACTGCTTATGCG<br>CTTTGTGTCACTAGCTATTGAAGGAAGCCCGAGGATGTTT

Supplementary Figures

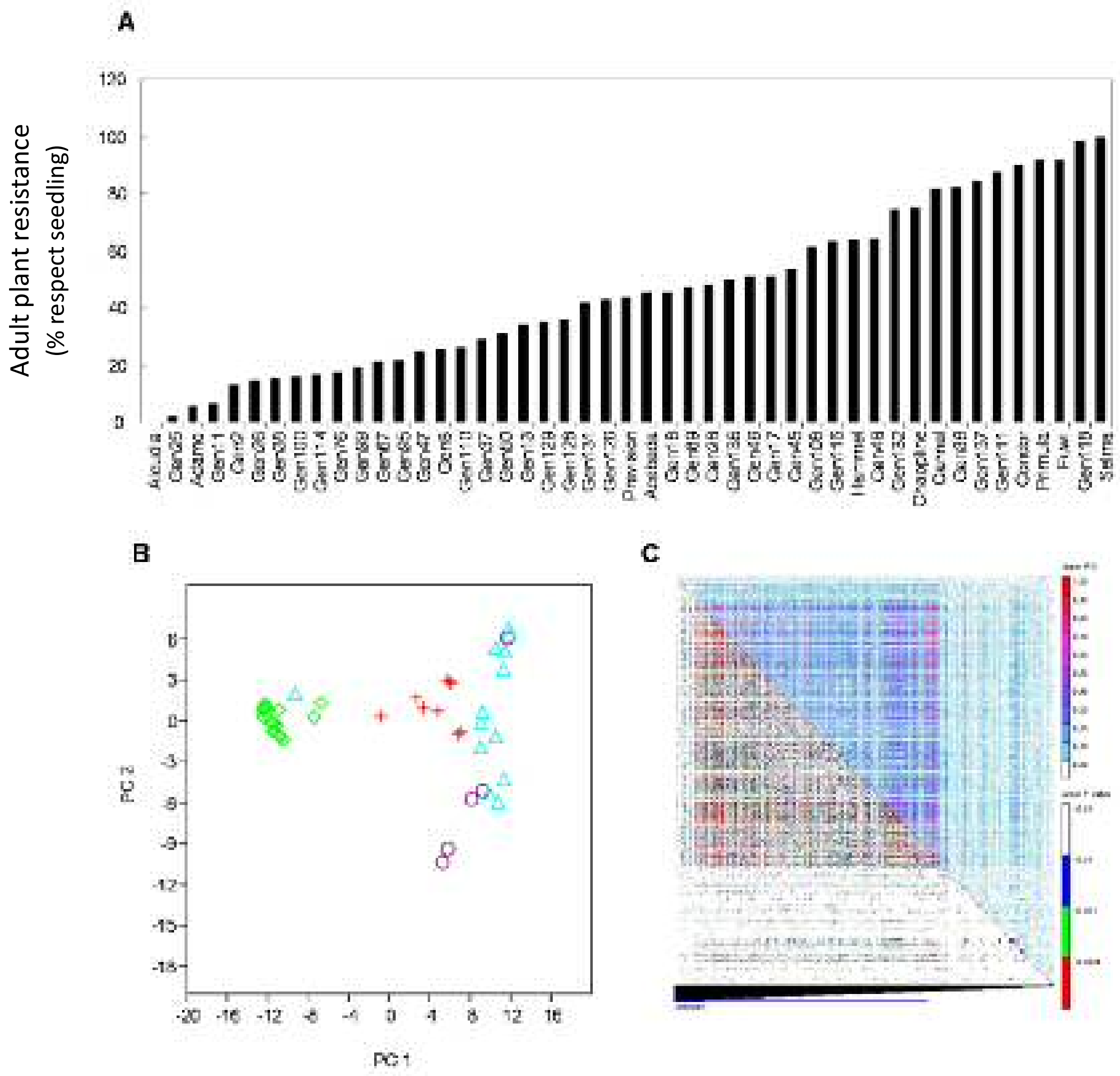

Supplementary Figure 1. A. Adult Plant resistance, measured as percentage of the seedling resistance in a subset of 54 oat accessions. **B.** Scatterplot of Principal Component Analysis scores of components 1 and 2 based on 1587 DArT and SSR markers used in this study in the oat subset. Represented are the genotypes belonging to cluster 1(red), cluster 2 (green), cluster 3 (violet) and cluster 4 (blue). **C.** Linkage disequilibrium matrix in the oat subset. Pair-wise LD values of polymorphic sites displaying r^2 above the diagonal and the corresponding p-values from rapid 1000 shuffle permutation test below the diagonal. Each cell represent the comparison of two pairs of marker sites with the color codes for the presence of significant LD. Colored bar code for the significance threshold levels in both diagonals is shown.

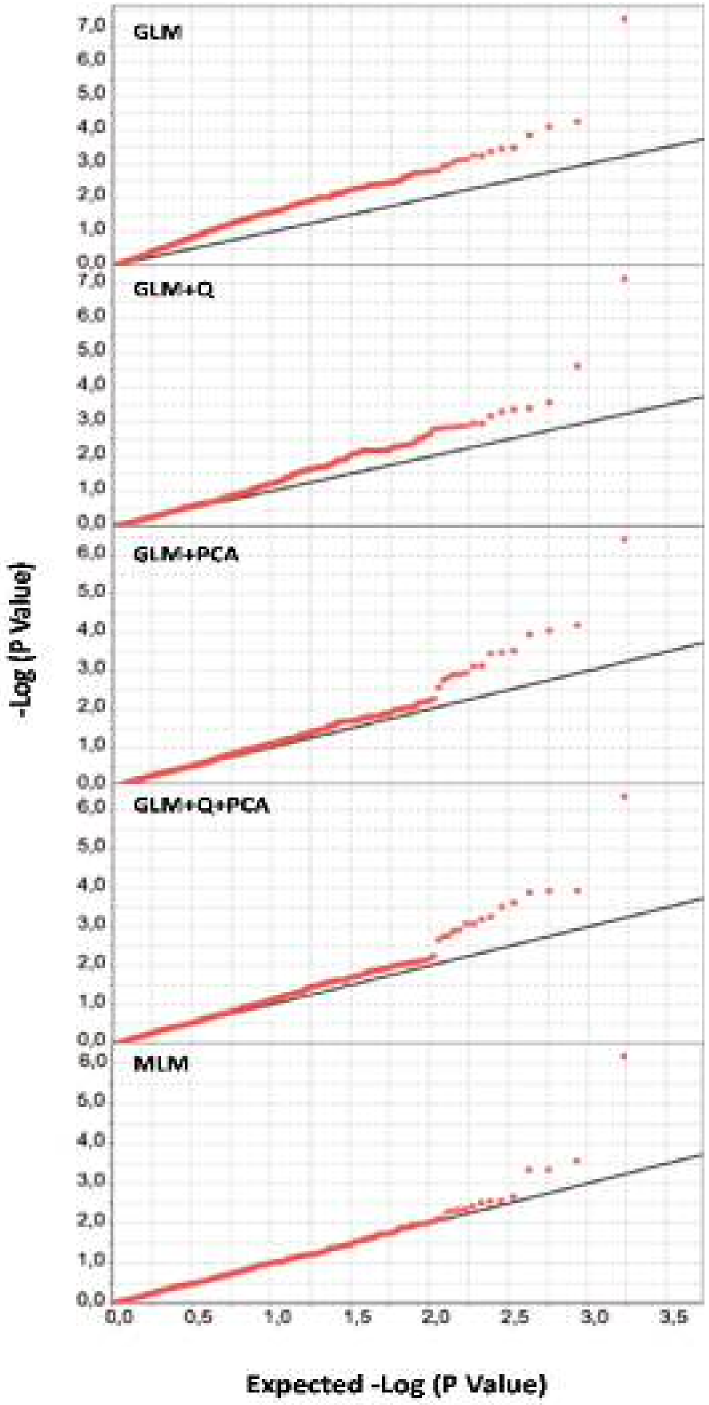

Supplementary Figure 2. Distribution of p values for the different models used in this study for the association between markers and rust resistance. Axes represented the expected p values versus the observed p values in the negative log10 scale where the solid line represent the null expectation (absence of type I errror).

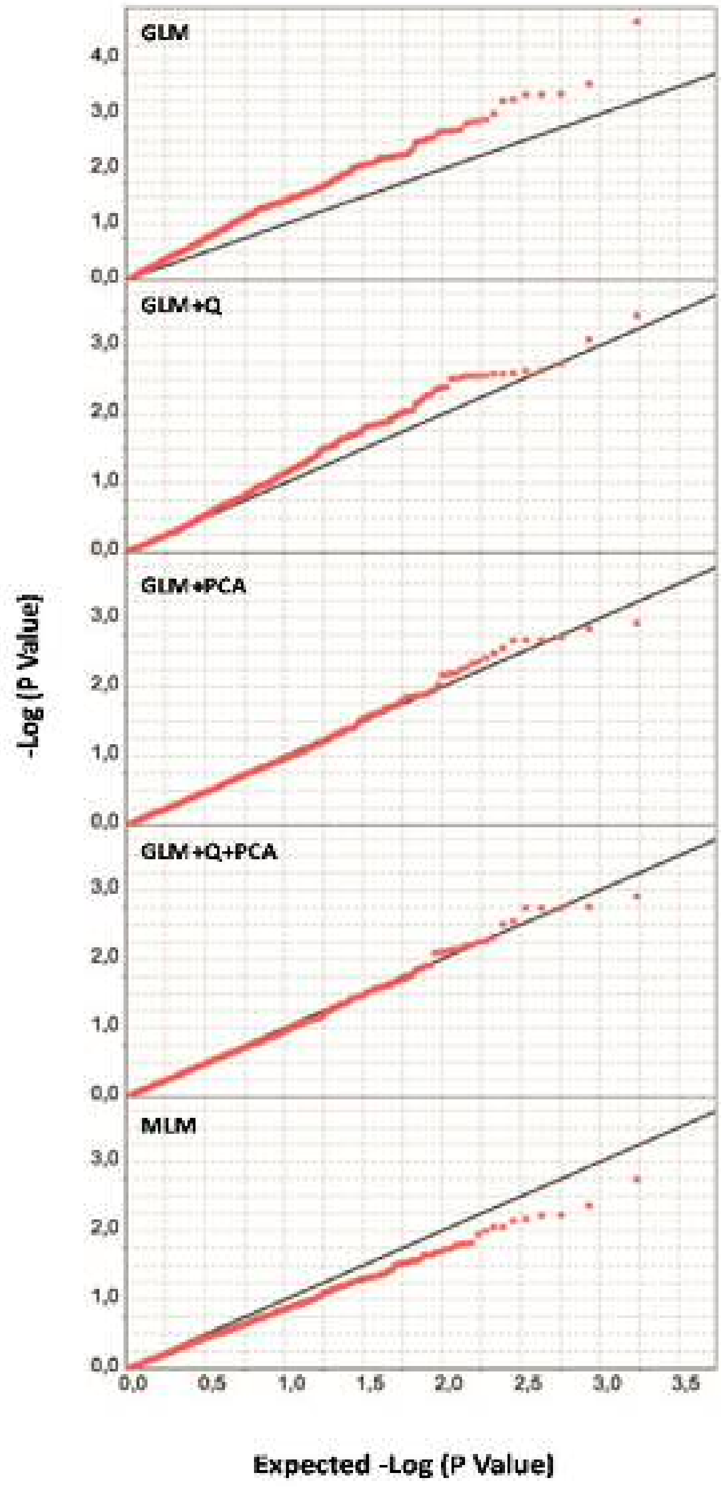

Supplementary Figure 3. Distribution of *p* values for the different models used in this study for the association between markers and powdery mildew resistance at seedling stage. Axes represented the expected *p* values versus the observed *p* values in the negative $\log_{10}$ scale where the solid line represent the null expectation (absence of type I errror).

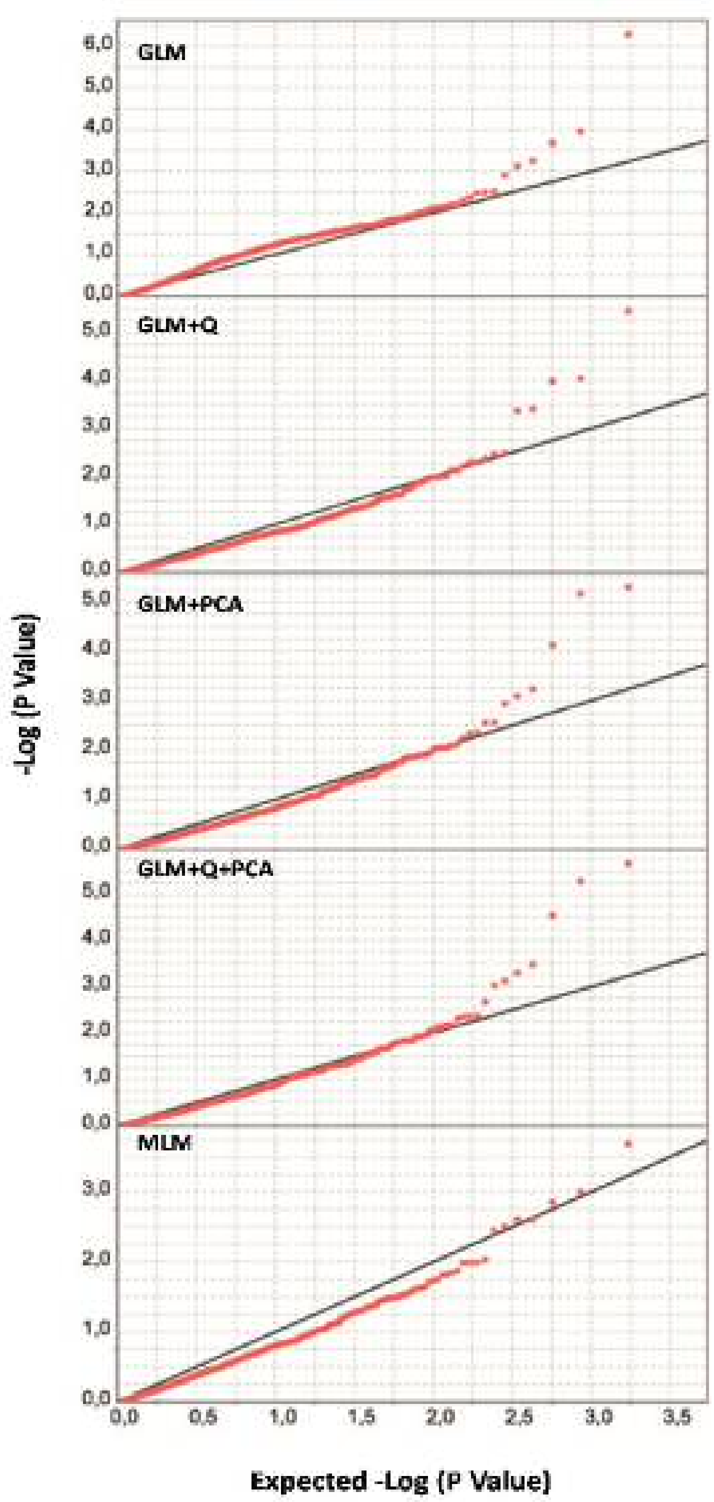

Supplementary Figure 4. Distribution of *p* values for the different models used in this study for the association between markers and powdery mildew resistance at adult plant stage. Axes represented the expected *p* values versus the observed *p* values in the negative $\log_{10}$ scale where the solid line represent the null expectation (absence of type I errror).

Marker oPt-11795

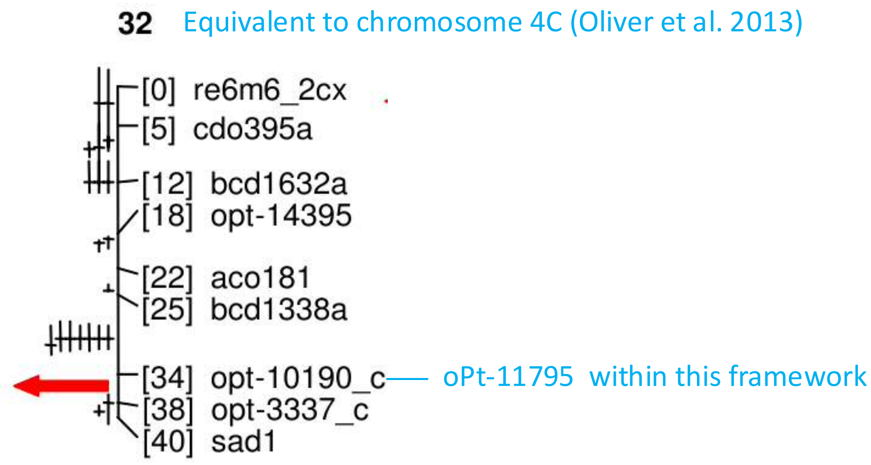

Marker MAMA5

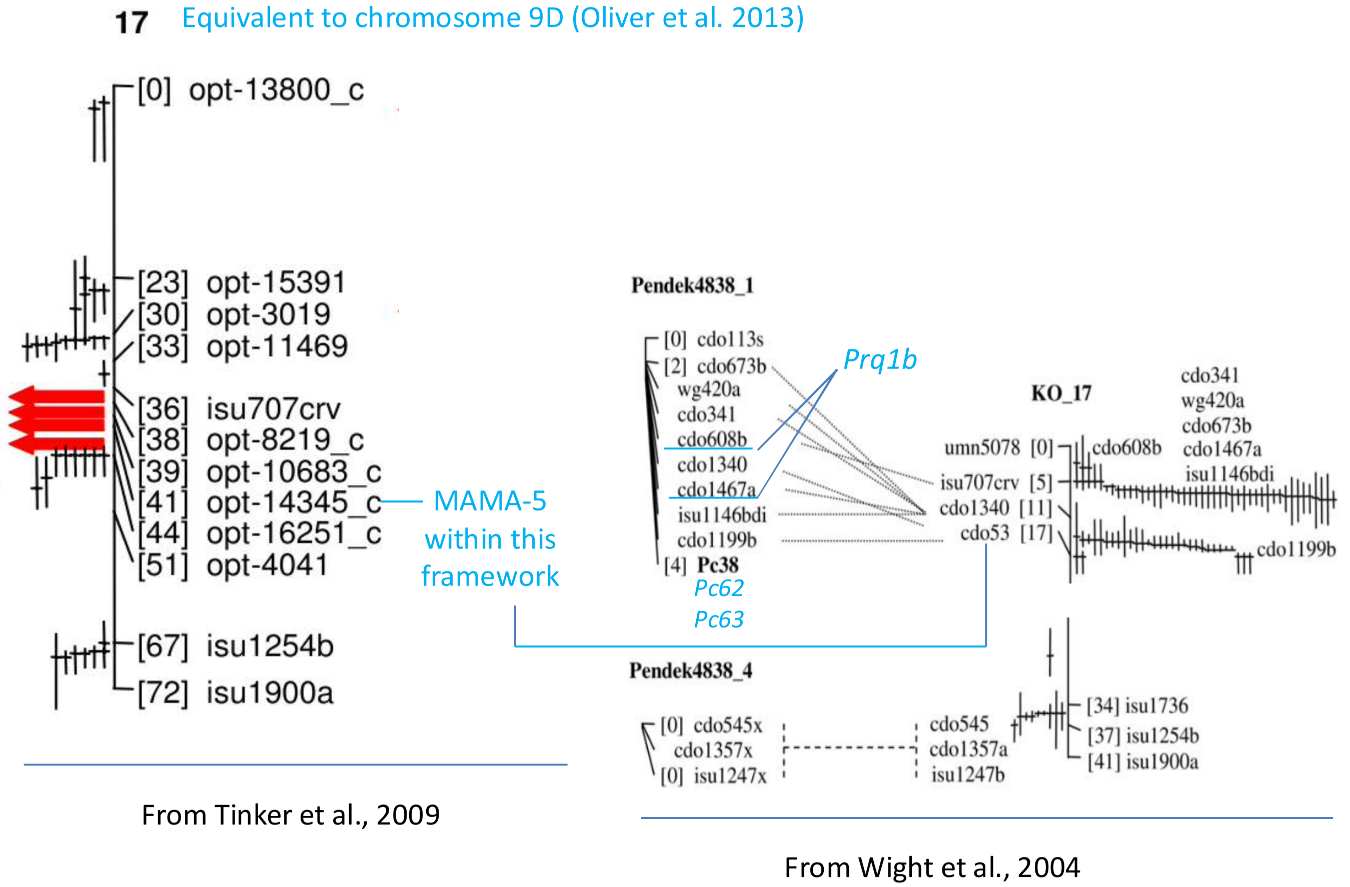

Supplementary Figure 5. Chromosomal organization of the different markers highlighted in this study according to localisations of previously reported markers. Cont.

Supplementary Figure 5. Cont

Marker AME176

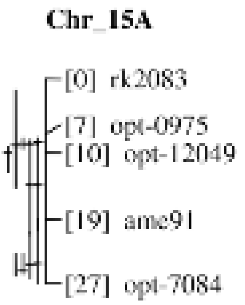

AME176 within this framework

From He et al., 2013

Marker oPt-14317

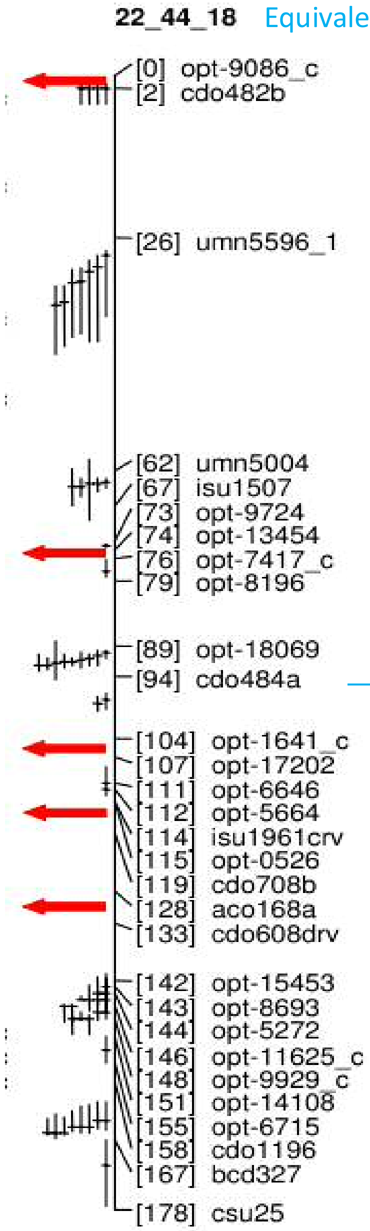

Equivalent to chromosome 19A (Oliver et al. 2013)

oPt-14317 and AM102 within this framework

From Tinker et al., 2009

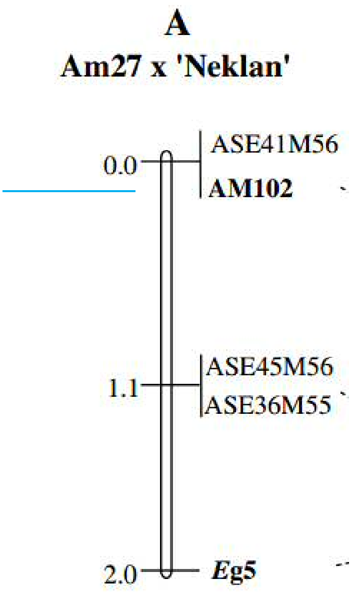

From Yu and Herrmann, 2006

Marker oPt-5014

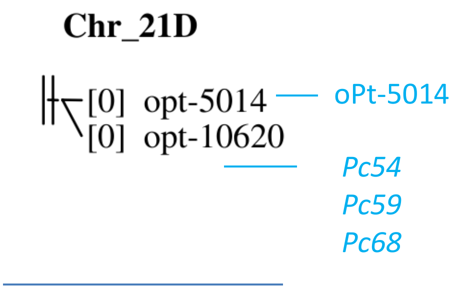

From He et al., 2013

Supplementary Figure 5. Cont
